# Type 1 Diabetes Prevention in NOD Mice by Targeting DPPIV/CD26 Is Associated with Changes in CD8^+^T Effector Memory Subset

**DOI:** 10.1371/journal.pone.0142186

**Published:** 2015-11-10

**Authors:** Núria Alonso, María Teresa Julián, Jorge Carrascal, Roger Colobran, Irma Pujol-Autonell, Silvia Rodriguez-Fernández, Aina Teniente, Marco Antonio Fernández, Antoni Miñarro, María Carmen Ruiz de Villa, Marta Vives-Pi, Manel Puig-Domingo

**Affiliations:** 1 Department of Endocrinology and Nutrition, Hospital Germans Trias i Pujol, Badalona, Department of Medicine, Autonomous University of Barcelona, Barcelona, Spain; 2 Immunology Department, Germans Trias i Pujol Research Institute, Autonomous University of Barcelona, Badalona, Spain; 3 Service of Immunology, Vall d’Hebron Research Institute, Vall d'Hebron University Hospital, Barcelona, Spain; 4 Cytometry Unit, Germans Trias i Pujol Research Institute, Badalona, Spain; 5 Department of Statistics, Faculty of Biology, University of Barcelona, Barcelona, Spain; 6 CIBER of Diabetes and Associated Metabolic Diseases (CIBERDEM). Instituto de Salud Carlos III (ISCIII), Madrid, Spain; Children's Hospital Boston/Harvard Medical School, UNITED STATES

## Abstract

CD26 is a T cell activation marker consisting in a type II transmembrane glycoprotein with dipeptidyl peptidase IV (DPPIV) activity in its extracellular domain. It has been described that DPPIV inhibition delays the onset of type 1 diabetes and reverses the disease in *non-obese diabetic* (NOD) mice. The aim of the present study was to assess the effect of MK626, a DPPIV inhibitor, in type 1 diabetes incidence and in T lymphocyte subsets at central and peripheral compartments. Pre-diabetic NOD mice were treated with MK626. Diabetes incidence, insulitis score, and phenotyping of T lymphocytes in the thymus, spleen and pancreatic lymph nodes were determined after 4 and 6 weeks of treatment, as well as alterations in the expression of genes encoding β-cell autoantigens in the islets. The effect of MK626 was also assessed in two *in vitro* assays to determine proliferative and immunosuppressive effects. Results show that MK626 treatment reduces type 1 diabetes incidence and after 6 weeks of treatment reduces insulitis. No differences were observed in the percentage of T lymphocyte subsets from central and peripheral compartments between treated and control mice. MK626 increased the expression of CD26 in CD8^+^ T effector memory (T_EM_) from spleen and pancreatic lymph nodes and in CD8^+^ T cells from islet infiltration. CD8^+^T_EM_ cells showed an increased proliferation rate and cytokine secretion in the presence of MK626. Moreover, the combination of CD8^+^ T_EM_ cells and MK626 induces an immunosuppressive response. In conclusion, treatment with the DPPIV inhibitor MK626 prevents experimental type 1 diabetes in association to increase expression of CD26 in the CD8^+^ T_EM_ lymphocyte subset. *In vitro* assays suggest an immunoregulatory role of CD8^+^ T_EM_ cells that may be involved in the protection against autoimmunity to β pancreatic islets associated to DPPIV inhibitor treatment.

## Introduction

Type 1 diabetes (T1D) results from the progressive destruction of insulin-producing pancreatic β-cells by CD4^+^ and CD8^+^ T cells [1]. Most self-reactive T cells are deleted by central tolerance mechanisms in the thymus; however even if central tolerance is highly efficient, a number of self-reactive cells escape from this barrier. In the *non-obese diabetic* (NOD) mouse, which spontaneously develops autoimmune T1D similar to the human disease, central and peripheral tolerance defects have been described [2].

CD26 is a type II transmembrane glycoprotein with dipeptidyl peptidase IV (DPPIV) activity in its extracellular domain. CD26 is constitutively expressed on the surface of many cell types, including immune cells [3], and a soluble form is also present in plasma. CD26 has been extensively characterized and multifunctional effects have been demonstrated [4]. The protein has at least 5 functions: a) serine protease, b) receptor for adenosine deaminase and CD45, c) co-stimulatory protein, d) adhesion molecule for collagen and fibronectin and e) involvement in apoptosis [5]. The most prominent and well-known biological properties are those related to signal transduction ability as a co-stimulatory molecule and proteolysis [6]. CD26 is preferentially expressed on a specific population of T lymphocytes, the subset of CD4^+^CD45RO^+^ memory T cells, and is up-regulated following T cell activation [7]. The enzymatic activity of CD26 appears to be very important in enhancing cellular responses to external stimuli being necessary for its co-stimulatory function [8]. It has recently been described that caveolin-1 in antigen presenting cells (APCs) is a binding protein for CD26. Their interaction results in the up-regulation of CD86 in the APC and enhances T cell co-stimulation [9]. The same group demonstrated that the catalytic site of CD26 is located in the pocket structure involved in the caveolin-1 scaffolding domain.

In CD4^+^ T cells, high CD26 cell surface expression correlates with the production of Th1-type cytokines and high transendothelial migratory activity, while CD26^+^ T-helper cells stimulate antibody synthesis in B cells [10]. In CD8^+^ T cells, CD8^+^CD26^bright^ T cell subset has recently been characterized as early effector memory T cells and reported to exert a cytotoxic effect, preferentially via B cells, TNF-α, IFN-γ and the Fas ligand [11]. In T1D patients, CD26 expression is reduced in the peripheral blood of CD8^+^ T cell subset [12]. CD26 is also thought to be a thymic maturation marker and its impairment has remarkable effects on lymphocyte composition, memory T cell generation and thymic emigration patterns [13,14].

Inhibition of DPPIV/CD26 suppresses antigen-stimulated T cell proliferation and cytokine production, thus suggesting a potential application for DPPIV inhibitors as immunomodulatory drugs in autoimmune diseases [15]. In animal models of autoimmune diseases, such as experimental autoimmune encephalomyelitis, *in vivo* inhibition of CD26 delays and attenuates the severity of the disease [16]. In this model, DPPIV inhibitors suppress IFN-γ, IL-4 and TNF-α production by antigen-stimulated CD4^+^ T cell clones [7]. In animal models of T1D, pre-treatment with a DPPIV inhibitor (sitagliptin) improves islet transplantation in both streptozotocin-induced diabetic mice [17] and in NOD mice [18]. In the latter, sitagliptin protects the islet graft through the modulation of CD4^+^ T cell migration and reduction of insulitis [19]. Moreover, DPPIV inhibition delays the onset of T1D [20] and reverses the disease in NOD mice [21]. Latent autoimmune diabetes in adults (LADA) is a form of less severe autoimmune diabetes than T1D. It has been recently reported that treatment with a DPPIV inhibitor in LADA patients attenuates the rate of decline of C-peptide levels, thus contributing to the maintenance of β-cell function [22]. Also, in the clinical setting, it has been described that the risk of autoimmune diseases is lower in those type 2 diabetes (T2D) patients who are being treated with a DPPIV inhibitor [23].

To our knowledge, the potential for modification of several T lymphocyte subsets from both the thymus and periphery with a DPPIV inhibitor in NOD mice, and its possible contribution to immune tolerance, have not been previously studied. The present work evaluates the effect of the DPPIV inhibitor MK626 in experimental T1D incidence and in thymic and peripheral T lymphocyte subsets, as well as functional mechanisms of action involved in β-cell tolerance reestablishment.

## Materials and Methods

### Ethics Statements

*In vivo* experiments were performed in strict accordance with the recommendations of the Guide for the Care and Use of Laboratory Animals of the Generalitat de Catalunya, Catalan Government and the Principles of laboratory animal care (NIH pub.85–23 revised 1985; http://grants1.nih.gov/grants/olaw/references/phspol.htm). The protocol was approved by the Committee on the Ethics of Animal Experiments of the Germans Trias i Pujol Research Institute (Permit number: DMAH5928).

### Mice

Wild-type NOD mice were obtained from our colony established with mice from the Jackson Laboratory (Bar Harbor, ME, USA). Only females were used for this study. Mice were kept under specific pathogen-free conditions in a 12 h dark/12 h light cycle with food and water *ad libitum*. At the end of the experiments, mice were sacrificed by cervical dislocation.

### Reagents and diets

MK626, a DPPIV inhibitor analog of des-fluoro-sitagliptin (kindly donated by Richard D. Carr, MSD Research Laboratories, New Jersey, USA), was administered daily in the diet (21 mg/kg, Research Diets, Inc. New Brunswick, NJ) for *in vivo* studies. Moreover, MK626, from the same source as for *in vivo* experiments, was used for *in vitro* studies.

### *In vivo* effect of MK626 treatment

NOD/LtJ mice were placed on either a normal chow diet (Research Diets, Inc. New Brunswick, NJ) or a diet containing the DPPIV inhibitor MK626. To determine the effect of MK626 in lymphocyte subsets, pre-diabetic mice (6–8 weeks of age) were treated during 4 and 6 weeks (5 mice/time-point). As control, untreated mice were used (5 mice/time-point). Spleen, pancreatic lymph nodes (PLNs) and pancreas were harvested for immunological analyses. All experiments were replicated twice.

To determine the *in vivo* effect of MK626 in T1D development, 15 NOD mice at 6–8 weeks of age were fed an MK626-containing diet. A control group of NOD mice (n = 15) was fed a normal chow diet. Mice were followed for diabetes onset until they reached 30 weeks of age. Incidence of the disease was determined at the end of the follow-up period.

Mice were monitored daily for urine glucose using Glucocard strips (Menarini, Barcelona, Spain). Mice with glycosuria were confirmed new-onset diabetic when successive blood glucose level was higher than 300 mg/dl after fasting for 2 hours (AccuCheck, Roche Diagnostics, Indianapolis, IN).

### DPPIV activity assessment

Plasma DPPIV enzymatic activity in mice receiving MK626 treatment and in control group was assessed by continuous fluorometric reaction (Edmonson SD, et al 2006) at 2, 4 and 6 weeks after treatment initiation (4 mice in each group/time-point). Briefly, the substrate Gly-Pro-Aminomethylcoumarin (AMC), containing a proline in a position cleaved by DPPIV, releases pro-AMC, generating a high fluorescent emission (RFU). The reaction substrate contains 50% plasma, 50 μM Gly-Pro-AMCand buffer (100 mMHepes pH 7.5, 0.1 mg/ml BSA) in a total volume of 50 μl. AMC liberation was monitored at 460 nm at 37°C (Viroskand Fluorometer, Thermo Electron, Germany). Data are reported as percent of inhibition.

### Insulitis score

The degree of islet infiltration by leukocytes (insulitis) was analyzed at 2, 4 and 6 weeks after treatment. Briefly, pancreases from 4 mice in each group were snap frozen in an isopentane/cold acetone bath. Cryosections of 5 μm were obtained at 5 non-overlapping levels. The sections were stained with hematoxylin and eosin (H&E) and analyzed by two independent observers. Each observer assessed a minimum of 40 islets per animal. Insulitis was scored as described elsewhere [24]: 0, no insulitis; 1, peri-insular; 2, mild insulitis (<25% of the infiltrated islet); 3, moderate insulitis (25–75% of the infiltrated islet); 4, severe insulitis (>75% islet infiltration).

### Tissue processing steps

Spleens and thymi were obtained at 4 and 6 weeks after treatment, whereas PLNs were obtained at 4 weeks. Thymic tissue was processed by enzymatic digestion. Briefly, small pieces of tissue were cut with a sterile blade, placed in 2% FCS-RPMI + 2 mg/ml Collagenase P (Boehringer Manheim, IN, USA) and incubated for 20 minutes at 37°C. The thymocyte suspension was washed and filtered using a 70-μm strainer and then re-suspended in Stain Buffer (BD Bioscience, San Jose, CA). The spleen was processed by mechanical disruption and mononuclear cells were isolated from the cell suspension by density gradient centrifugation using Lympholyte Mouse (Cedarlane Labs, Burlington, Canada), at 800g for 20 minutes at RT, and cells were then washed and filtered using a 70-μm strainer. Cells were re-suspended in Stain Buffer (BD Bioscience). PLNs were removed from mice and put in Phosphate Buffer. Then, PLNs were mechanically disrupted and re-suspended in a single cell suspension before the staining.

### Flow Cytometry

Thymocytes and peripheral T lymphocyte subsets from NOD, with or without MK626 treatment, were stained using an optimized panel of fluorochrome-conjugated monoclonal antibodies (mAb) (shown in Table 1). Specifically for T cell regulatory subset, T cells from spleen and PLNs were stained with anti-CD4-APCCy5.5 (BD), anti-CD25-PerCP (BD) and anti-FoxP3-eF450 (EBiosciences). Also T cells were stained with anti-CD8-V500 (BD), anti-PD1-PeCy7 (BD) and anti-CD122-biotin (BD). SA-APC (BD) was used as a secondary antibody. Analyses were run on a FACS Canto II flow cytometer (BD).

**Table 1:** List of mAb used for the analysis of lymphocyte subsets. BD (BD Biosciences, San Jose, CA); eB (eBioscience, San Diego, CA); DN: double negative; DP: double positive; SP: single positive; T _CM_: central memory T cell; T _EM_: effector memory T cell; CD26 expression was assessed in all lymphocyte subsets.

Lymphocyte subset	Phenotype	Marker
DN	CD3^+^CD4^-^CD8^-^	CD3-V450 (BD)CD4-APC-Cy7 (BD)CD8a-V500 (BD)CD26-PerCP-Cy5.5 (eB)CD25-PE (BD)CD62L-PerCP-Cy5.5 (BD)CD44-FITC (BD)FoxP3-APC (eB)PD1-PeCy7 (eB)CD122-biotin (eB)
DP	CD3^+^CD4^+^CD8^+^	
SP CD4^+^	CD3^+^CD4^+^CD8^-^	
SP CD8^+^	CD3^+^CD4^-^CD8^+^	
DN1	CD8^-^CD4^-^CD44^high^CD25^-^	
DN2	CD8^-^CD4^-^CD44^high^CD25^high^	
DN3	CD8^-^CD4^-^CD44^-^CD25^high^	
Naïve	CD3^+^CD4^+^/CD8^+^CD44^low^CD62L^+^	
T _CM_	CD3^+^CD4^+^/CD8^+^CD44^high^CD62L^+^	
T _EM_	CD3^+^CD4^+^/CD8^+^CD44^high^CD62L^neg^	
CD4^+^T regulatory cells	CD4^+^CD25^+^FoxP3^+^	
CD8^+^T regulatory cells	CD8^+^PD1^+^CD122^+^	

### GLP-1 determination

Plasma was obtained at 2, 4 and 6 weeks after treatment (4 mice/time-point). GLP-1 was determined using the active GLP-1 ELISA kit (Millipore, Billerica, MA), specific for GLP-1 (7–36) amide form, the main form of circulating biologically active GLP-1.

The experiment was replicated twice.

### Plasma TGF-β concentration

TGF-β levels were quantified in serum at 4 and 6 weeks (5 mice/time-point) in MK626-treated and control NOD mice, using a specific Human/Mouse TGF-β1 ELISA Ready-SET-Go kit (e-Bioscience, USA). The experiment was replicated twice.

### Phenotyping of pancreatic islet infiltrate

To characterize islet infiltrating leukocytes and to determine their CD26 expression, pancreases from treated (n = 4) and control NOD mice (n = 4) were obtained 4 weeks after treatment. The experiment was replicated twice. Briefly, pancreases were perfused with collagenase (Collagenase CLS-4; Worthington Biochemical, Lakewood, NJ) through the common bile duct as described [25]. Pancreases were removed and incubated at 37°C for 15 min. After digestion, collagenase was neutralized with HBSS medium and the digested tissue was placed into a petri dish. Islets were hand-picked, mechanically homogenized and filtered with a 70 μm cell strainer to obtain a single cell suspension. For the identification of cell subsets, cell suspension was stained with a primary antibody Rat anti-mouse CD8 (clone Lyt-2.2; MyBiosource, San Diego, CA). After a single wash, a secondary antibody Goat anti-Rat FITC (SouthernBiotech, Birmingham, AL) was used. Finally, a third stage staining was performed with an anti-CD3 V450 (BD), anti-CD4 APC-Cy7 (BD) and anti-CD26 PE antibody (BioLegend, San Diego, California).

### T lymphocyte stimulation assay

Spleens were removed from treated (n = 5) and control mice (n = 5) and erythrocytes were lysed in 0.87% ammonium chloride. From each group 3x10^7^ splenocytes were firstly stained with biotinylated anti-CD62L (clone MEL14-H2.100, Miltenyi Biotech) and with the Pan T Isolation Kit II (Miltenyi, 130-095-130). Splenocytes were passed through a Magnetic LS MACS column (Miltenyi 130-042-401) and the unbound fraction corresponding to Effector memory (T_EM_) T lymphocytes was isolated. A second stain was performed to purify CD8^+^ T_EM_ lymphocytes. Then, cells obtained were stained with an anti-CD4 antibody, clone L3T4 (Miltenyi Biotech) and passed through another LS column. The unbound eluted fraction, corresponding to the CD8^+^ T_EM_ lymphocytes subset, was finally used for the proliferation assays. Stimulus consisted of 1 μg/ml of anti-CD3 monoclonal antibody (clone 145-2C11, BD Bioscience) plated 24 hours before the assay. 5x10^4^ CD8^+^ T_EM_ lymphocytes from treated or control mice were cultured in round bottom 96 wells during 72 hours. Cells were pulsed with 1 μCi of [3H]thymidine in the last 18 hours of the assay. Cells were harvested (Harvester 96, Tomtec Inc., Hamden, CT) and analyzed using a scintillation counter (1450 Microbeta, TriluxWallac, Turku, Finland). T cell proliferation was expressed as counts per minute (c.p.m). After 54 hours of T lymphocyte stimulation, supernatants were collected and cytokine production was assessed by flow cytometry using the Bead Array-CBA kit (BD Bioscience), which measures IL-2, IL-4, IL-6, IL-10, IL-12, IL-17, IFN-γ and TNF-α concentration. Data were analysed using the CBA software. TGF-β levels were quantified using a specific Human/Mouse TGF-β1 ELISA Ready-SET-Go kit (e-Bioscience, USA). The experiments were replicated twice.

### Biomarkers of β-cell regeneration by qRT-PCR

To determine if DPPIV inhibition allows specifically β-cell regeneration, we investigated whether MK626 alters the expression of genes encoding β-cell autoantigens. As control, *glucagon* gene expression was also evaluated to assess α-cell mass increase. CD45 (lymphocyte common antigen) expression was used as a marker of T-lymphocytic infiltrate. Total RNA was extracted using the kit RNeasy Micro (QIAGEN, Venlo, Limburg, The Nederlands) from whole pancreases from 5 control and 5 MK626-treated animals after 4 weeks of treatment. Prior to RNA isolation, cryosections of frozen pancreata were mechanically disaggregated when thawed. MinElute Spin Columns were used to extract RNA from samples following manufacturer's instructions. RNA integrity and concentration was measured using 2200 TapeStation (Agilent Technologies, Santa Clara, CA). cDNA synthesis reactions were carried out using random hexamers (0.5 μg/μl, BioTools, Valle de Tobalina, Madrid, Spain) and reverse transcriptase Moloney-murine-Leukaemia-virus (M-MLV) (200 U/μl, Promega, Madison, WI), and samples were incubated for 60 minutes at 37°C. Finally, qRT- PCR were performed under Taqman universal assay on the Light Cycler 480 system using the following TaqMan assays: *Insulin II* (*Ins2)* (Mm00731595_gH), *Islet amyloid polypeptide* (*Iapp)* (Mm00439403_m1), *Glucagon* (*Gcg*) (Mm01269055_m1) and *CD45* (Mm01293577_m1). House-keeping gene *Gapdh* (Mm99999915_g1) expression was also assessed in order to normalize the expression of the target genes to that of the housekeeping gene, as described in the relative quantification method (2^-ΔCp^) [26], and was referred as arbitrary units.

### T lymphocyte suppressive assay

MK626 was diluted in water and tested *in vitro* at 10 μM to determine the effect on the viability of splenocytes, as a previous step for the suppression assay. Briefly, splenocytes were obtained after mechanical disruption of spleen and cultured in presence of different concentrations of MK626. Viability was assessed at 2, 24 and 48 hours by annexin V-PE and 7-aminoactinomycin D labelling (7aad) (BD Pharmingen, San Diego, CA).

To assess whether MK626 increases CD8^+^ T_EM_ lymphocytes’ immunosuppressive function, the putative regulatory T lymphocytes and effector T lymphocytes obtained from non-diabetic NOD mice at 10–12 weeks of age were differentially labelled with intracellular fluorescent dyes CellTrace Violet (CTV, Molecular Probes, Molecular Probes, Invitrogen, Thermo Fisher Scientific Inc) and carboxyfluorescein diacetate, succinimidyl ester (CFSE, Molecular Probes, Invitrogen, Thermo Fisher Scientific Inc, Waltham, MA), respectively. Autologous suppression assays were established in 96-well plates with 4x10^4^ CFSE-labelled splenic T lymphocytes and 4x10^3^ autologous insulin-loaded mDCs, as antigen presenting cells (APCs), cocultured with CD8^+^ T_EM_ lymphocytes at different ratios (1:0.5, 1:1 and 1:2, being T cell:CD8 T_EM_), in presence or absence of MK626 (10μM). Splenic T lymphocytes were purified with the Pan T Isolation Kit II (Miltenyi) through a Magnetic LS MACS column (Miltenyi). CD8^+^ T_EM_ lymphocytes were purified as detailed before (see T lymphocyte stimulation assay). Insulin-loaded mature DCs (mDCs) were obtained as previously described [27]. Briefly, immature DCs (iDCs) were generated *in vitro* from bone marrow progenitors of NOD mice in culture medium containing GM-CSF (1000 U/ml; Prospec, Rehovot, Israel). iDCs were cocultured in presence of insulin (20μg/ml, Sigma) for 2 hours and stimulated with lipopolysaccharide (LPS, 100 ng/ml; Sigma) for 24 hours to obtain mDCs. Finally, after 3 days of coculture, cells were harvested and data were acquired by flow cytometry (FACSCanto II) and analysed using the FlowJo software (Tree Star, Ashland, OR). Dead cells were excluded from the analysis using 7aad (BD Pharmingen).

### Statistical analysis

Data analysis was performed by applying a three-way ANOVA for every continuous variable, controlling by group (two levels, control and treatment), week (two levels, week 4 and week 6) and replicate (two levels, replicate 1 and 2). The replicate was considered a block factor, not affecting any interaction including replicate factor; thus the group-per-week interaction was the only interaction factor considered. Observations with a Studentized residual (in absolute value) greater than 3 were considered outliers and were removed from the analysis. This situation usually affected just one individual. Post-hoc analyses using Tukey’s HSD were performed to detect differences between the combinations of factors per group and week. Otherwise, Mann-Whitney U-test was applied. Kaplan-Meier log-rank analysis was used to compare the incidence of diabetes between different groups. A p-value ≤ 0.05 was considered significant. Statistical analysis was performed using the Prism 5.0 software (GraphPad software Inc., San Diego, CA) and “R” software version 3.0 (www.rproject.org).

## Results

### DPPIV inhibitor MK626 reduces the incidence of diabetes and the insulitis score

We first evaluated the effect of an MK626-containing diet. The percentage of plasma DPPIV activity was reduced by more than 90% in the MK626-treated group, and this inhibition was maintained throughout the study period (week 4: 95.82%; week 6: 92.44%.), thus confirming that DPPIV activity was mostly inhibited in the MK626-administered mice (S1A Fig). Plasma levels of active GLP-1 displayed a biological increase in the MK626-treated mice, although statistically non-significant (S1B Fig).

**Fig 1:**
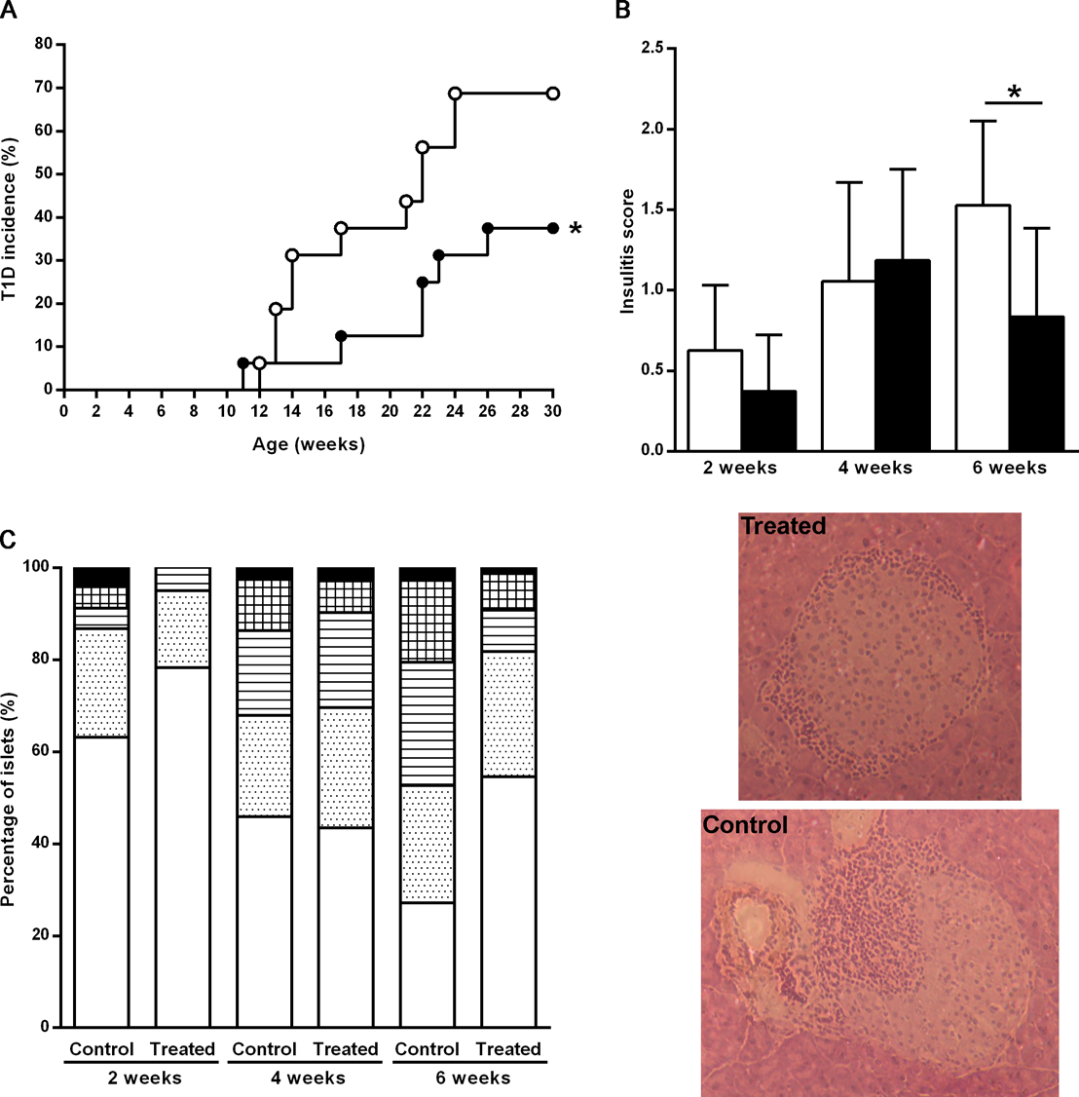
DPPIV inhibitor MK626 decreases T1D incidence and reduces the insulitis score in NOD mice. A) Cumulative incidence (percentage) of diabetes in NOD mice treated with MK626 (black circles, n = 15) and in the control group (white circles, n = 15). Significant differences were found when compared MK626-treated mice versus control group (*p≤0.05, Kaplan-Meier log-rank analysis). B) Insulitis score from treated and untreated mice at each time-point. Pancreases from 4 mice/group were analyzed by two independent observers at 2, 4 and 6 weeks after treatment. Insulitis score was significantly lower in treated mice than in controls after 6 weeks of treatment. Results are expressed as mean ± SEM (*p≤0.05, Mann-Whitney test). Lower panel: sections of pancreas from haematoxylin and eosin illustrating islets taken from a treated and control mice at 6 weeks. c) Percentage of islets in each of the five infiltration categories in treated and control mice at 2, 4 and 6 weeks: White = 0, no insulitis; Dotted = 1, peri-insular; Striped = 2, mild insulitis (<25% of the infiltrated islet); Squared = 3, moderate insulitis (25–75% of the islet infiltrated); Black = 4, severe insulitis (>75% islet infiltration).

At the end of the follow-up period of the incidence study at week 30, T1D incidence was significantly decreased in MK626-treated mice (37%) when compared with control mice (68%), (p = 0.05, Fig 1A). Thus, a 31% reduction in the incidence of diabetes was achieved in the treated group. In view of the reduced incidence of diabetes in MK626-treated mice, we next aimed to determine the impact of MK626 on insulitis score at 2, 4 and 6 weeks of treatment. The insulitis score was significantly lower in treated mice than in controls after 6 weeks of treatment (0.83 ± 0.27 vs. 1.52 ± 0.26, respectively; p = 0.022) (Fig 1B), whereas no differences were observed between groups at week 2 and at week 4 of treatment. Representative images of islets from haematoxylin and eosin-pancreatic cryostat sections from treated and control mice at 6 weeks after treatment are showed in the lower panel of Fig 1B. Moreover, analysis of the percentage of islets classified in each of the five infiltration categories showed that in 6 weeks-treated mice most of the islets remained free of insulitis or with peri-insulitis, whereas in the control group most of the islets were scored with a severe degree of insulitis (Fig 1C). Insulitis-free islets were more than 50% in MK626-treated mice at 6 weeks.

These results strongly suggest that DPPIV/CD26 inhibition by MK626 prevent T1D through impairment of islet autoimmune attack.

### MK626 treatment does not modify plasma TGF-β levels

**Fig 2:**
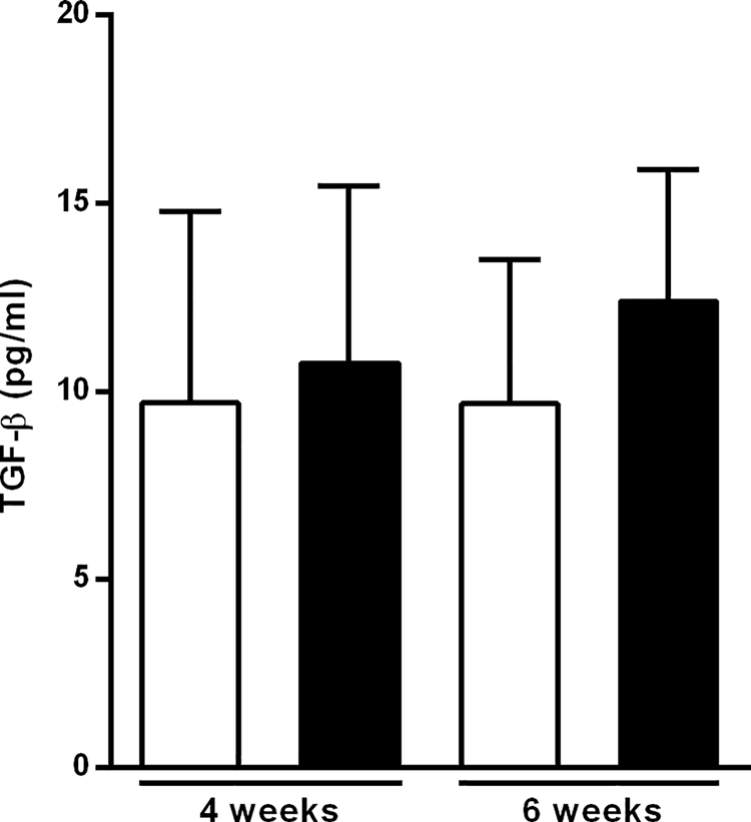
Serum TGF-β concentration from NOD mice after MK626 treatment. Serum level of TGF-β from control (white bars) and treated (black bars) after 4 and 6 weeks of treatment. Results are expressed as mean ± SD from 10 mice/time-point. No statistical differences were observed between groups (Mann-Whitney test).

The effect of MK626 on serum levels of TGF-β was evaluated at 4 and 6 weeks after treatment. Although plasma TGF-β levels displayed a biological increase in MK626-treated mice compared to control mice, the difference was not statistically significant at any of the study’s time-points (Fig 2).

### MK626 increases CD26 expression in CD8^+^ Effector memory T cells

To investigate the potential mechanisms by which the DPPIV inhibitor MK626 induced a reduction in T1D incidence and insulitis score, we aimed to determine whether treatment with MK626 was associated with changes in T cell subsets from both the thymus and peripheral compartments (spleen and PLNs) and/or in their expression of CD26, which could contribute to the development of immune tolerance.

#### 1. *Thymus*: Treatment with MK626 does not modify thymocyte subsets

Because CD26 has been shown to play a role in the differentiation and maturation of thymocytes, the effect of MK626 on the percentage of thymocytes subsets was next studied. No significant changes in the percentages of single positive (SP) CD4^+^ and SP CD8^+^, double positive (DP) CD4^+^CD8^+^ and double negative (DN) CD4^-^CD8^-^ (DN1, DN2, DN3) thymocytes were observed in MK626-treated mice compared to controls after 4 and 6 weeks of treatment (S2A Fig).

No differences were observed between treated and control mice in the mean fluorescence intensity (MFI) of the same subsets expressing CD26 (S2B Fig). Finally, no differences were observed between groups in the percentage and CD26 expression on natural regulatory T cells (Tregs) (CD4^+^CD25^+^FoxP3^+^) at any of the study’s time-points (S2C Fig). These results suggest that the reduction of insulitis and the decrease in the incidence of T1D observed in MK626-treated mice are not related to thymocyte subsets alterations in NOD mice.

#### 2. *Peripheral compartments*: Treatment with the DPPIV inhibitor MK626 increases the expression of CD26 on CD8^+^ Effector memory T cells from both the spleen and pancreatic lymph nodes

A. *Spleen*

First we aimed to ascertain whether treatment with MK626 had any effect on the percentage of CD4^+^ and CD8^+^ T lymphocytes within the CD3^+^ population. No differences were observed in the percentage of total CD4^+^ T cells nor in CD8^+^ T cells at any of the study’s time-points (4 and 6 weeks) between treated and control mice (S3A Fig). Moreover, the MFI for CD26 on CD4^+^ and CD8^+^ T lymphocytes was similar in the two groups (S3B Fig).

**Fig 3:**
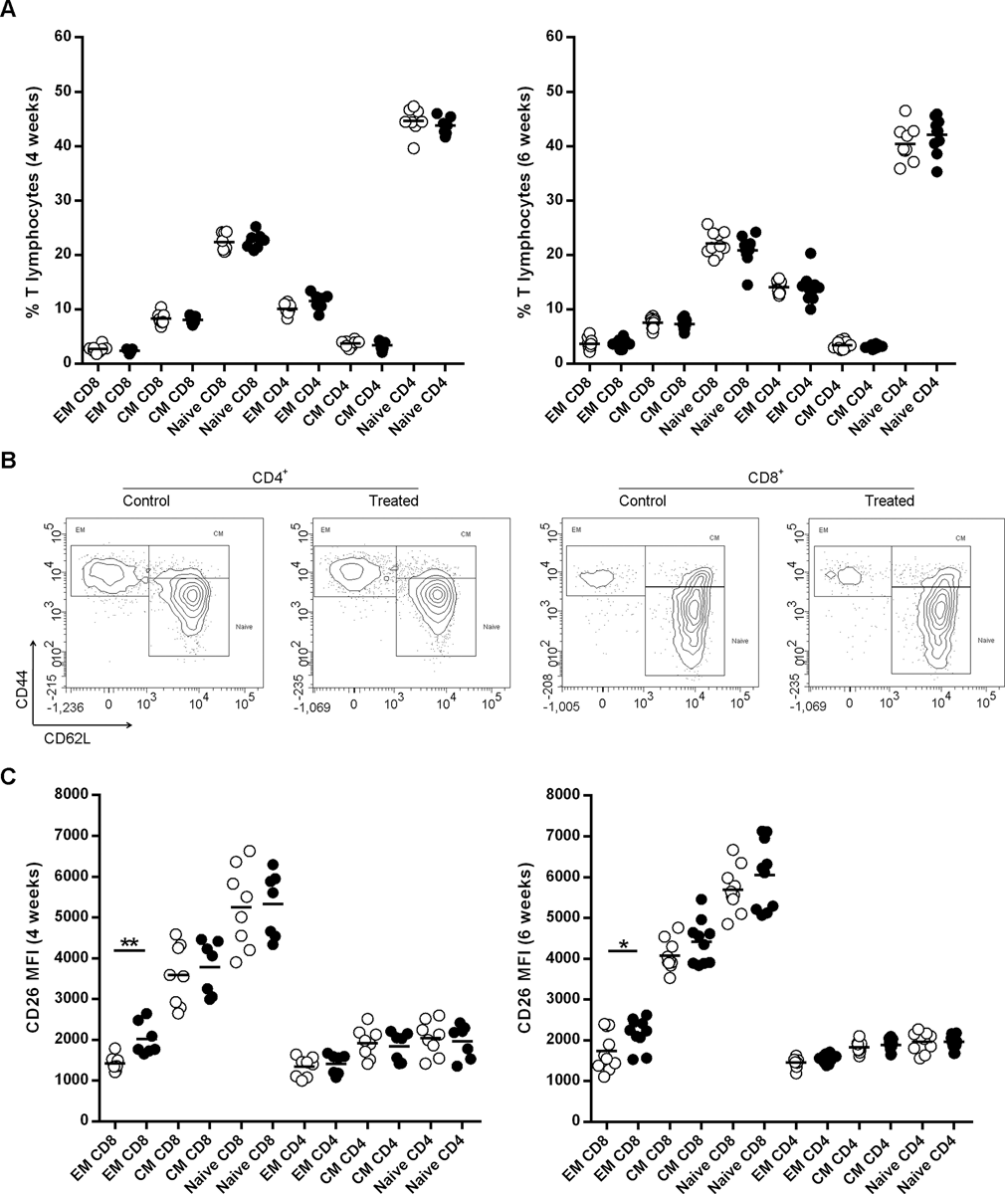
Effects of the DPPIV inhibitor MK626 on splenic T- lymphocytes. Splenocytes were obtained at 4 and 6 weeks after treatment. A) Percentage of T effector memory (EM)_,_ T central memory (CM) and naïve T lymphocytes (CD8^+^and CD4^+^) in control (white circles) and treated mice (black circles). B) Representative FACS plots of the naïve (CD3^+^CD4^+^/CD8^+^CD44^low^CD62L^+^), T_CM_ (CD3^+^CD4^+^/CD8^+^CD44^high^CD62L^+^) and T_EM_ (CD3^+^CD4^+^/CD8^+^CD44^high^CD62L^neg^) both for CD4^+^ and CD8^+^ T lymphocytes from spleen in treated and control mice at 4 weeks of treatment. C) MFI for CD26 expression on T_EM,_ T_CM_ and naïve T lymphocytes (CD8^+^ and CD4^+^) in control (white circles) and treated mice (black circles). Lines represent the mean of 8–10 mice. Comparison between treated and untreated mice showed an increased expression of CD26 on CD8^+^ T_EM_ cells at any of the study’s time-points. (*p<0.05, **p<0.01, three way Anova).

Next, naïve, central and effector memory T cell (T_CM_ and T_EM_) subsets were analyzed. No differences were observed in the percentage of naïve, T_CM,_ T_EM,_ either in the CD4^+^ or in the CD8^+^ T cell subsets after 4 and 6 weeks of treatment with MK626 (Fig 3A). A representative example of the multicolour flow cytometric analysis of naïve, T_CM_ and T_EM_ CD4^+^ and CD8^+^ T cell subsets in the spleen of control and treated mice at week 4 of treatment is shown in Fig 3B.

Then, we evaluated the CD26 expression on T cell subsets by MFI. The percentage of CD4^+^ and CD8^+^ T cells showing high density expression of CD26 (CD4^+^CD26^high^/CD8^+^CD26^high^) was not studied due to the fact that there was not a clear subset delimitation after cytometric analysis. After 4 and 6 weeks of treatment, the MFI of CD26 expression was significantly increased on CD8^+^ T_EM_ subset in NOD treated mice compared to controls (p = 0.002 at week 4; p = 0.03 at week 6) (Fig 3C). No differences were observed between treated and control mice at any of the study time-points in the CD26 expression on the CD4^+^ T_EM_ and naïve lymphocytes and T_CM_ subset (CD8^+^ and CD4^+^ subpopulations). Finally, MK626 treatment did not modify the percentage and MFI of CD26 expression in CD4^+^ Tregs (CD4^+^CD25^+^FoxP3^+^) (S3C Fig) nor in CD8^+^ Tregs (CD8^+^ CD122^+^PD-1^+^) at any of the analyzed time-points (data not shown).

**Fig 4:**
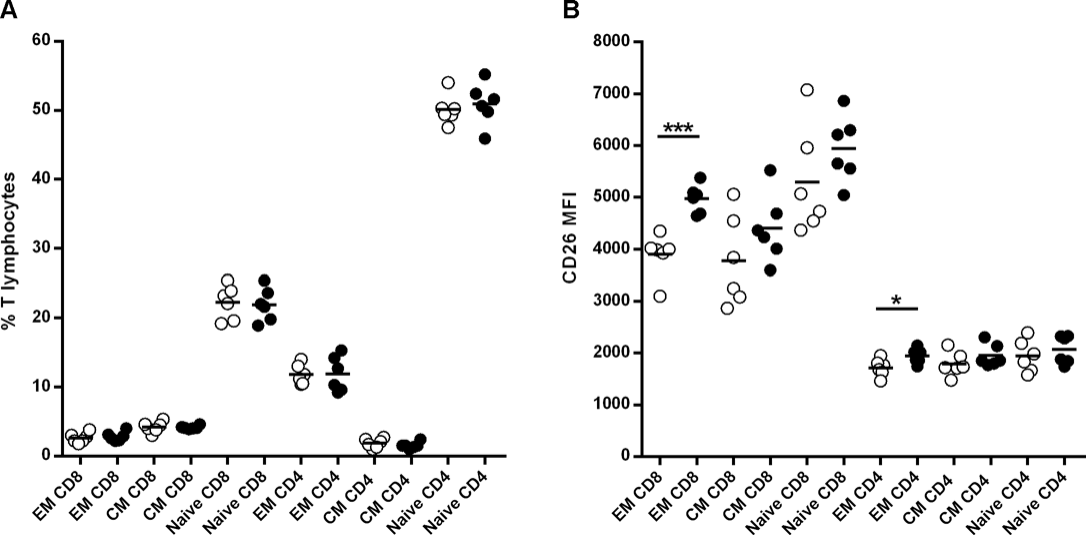
Effects of the DPPIV inhibitor MK626 on T lymphocytes from pancreatic lymph nodes (PLNs). T cells from PLNs were obtained from control (white circles) and treated (black circles) NOD mice at 4 weeks after treatment. A) Percentage of T effector memory (EM)_,_ T central memory (CM) and naïve T lymphocytes (CD8^+^and CD4^+^). B) MFI for CD26 expression on T_EM,_ T_CM_ and naïve T lymphocytes (CD8^+^and CD4^+^). The expression of CD26 was significantly increased on CD4^+^ T_EM_ and CD8^+^ T_EM_ cells in MK626-treated mice when compared to controls. Lines represent the mean of 6 mice (*p<0.05, ***p<0.001, three way Anova).

B. *Pancreatic lymph nodes* In addition, we investigated whether data observed in spleen was replicated in PLNs after 4 weeks of treatment. No differences were observed in the percentage of T cell subsets (naïve, T_CM,_ T_EM_) between MK626-treated mice and controls (Fig 4A). In relation to CD8^+^ T cells, similar to the findings in spleen, we observed that the MFI of CD26 expression was significantly higher in the CD8^+^ T_EM_ subset in NOD treated mice compared to controls (p = 0.0004) (Fig 4B). Regarding CD4^+^ T cells, an increased expression of CD26 in T_EM_ subset was observed in the PLNs lymphocytes (p = 0.03). There were no significant differences between groups in CD26 expression on the naïve T cell and T_CM_ subset (CD8^+^/CD4^+^), and neither in the conventional Tregs subsets (CD8^+^/CD4^+^) (data not shown).

Taken together, results obtained show that treatment with the DPPIV inhibitor MK626 increases the expression of CD26 in the CD8^+^ T_EM_ cell subset, both in spleen as well as in PLNs without affecting their percentage.

### MK626 increases CD26 expression on CD8^+^ lymphocytes from pancreatic islet infiltrate

**Fig 5:**
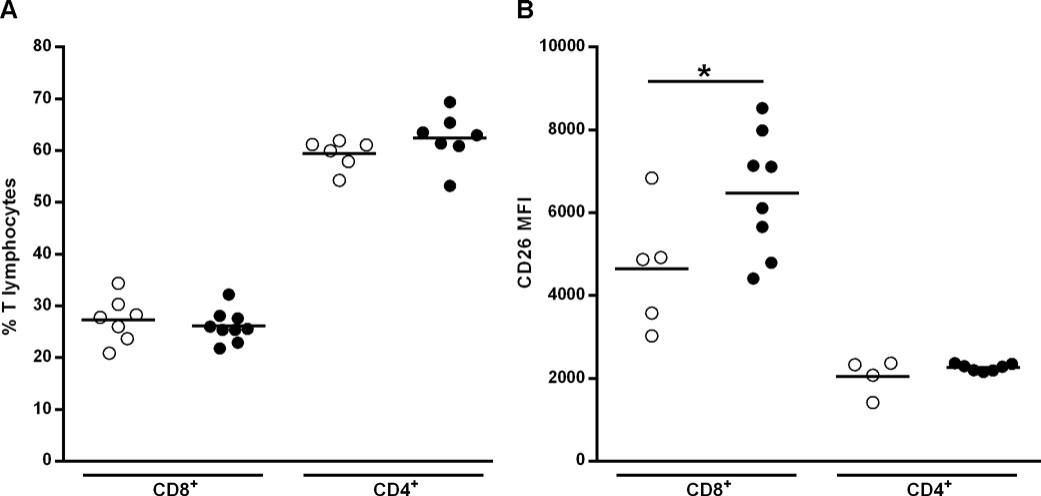
Percentage of T lymphocytes and CD26 membrane expression in the leukocytic infiltrate of mice treated 4 weeks with MK626. A) Percentage of CD8^+^ and CD4^+^ T lymphocytes infiltrating pancreatic islets in untreated (white circles) and treated (black circles) mice assessed by flow cytometry. Lines represent the mean of 6–9 mice. No statistical differences were observed between groups (Mann-Whitney test). B) Expression of CD26 determined by flow cytometry (MFI) in CD8^+^ and CD4^+^ T lymphocytes from control (white circles) and treated (black circles) mice. Lines represent the mean of 4–8 mice. (*p≤0.05, Mann-Whitney test).

To assess the mechanism through which DPPIV inhibitor treatment reduces insulitis and prevents T1D in NOD mice, the changes observed in T lymphocyte subsets obtained from the pancreatic islet infiltrate were analyzed. Firstly, we evaluated whether treatment with MK626 had any effect on the percentage of CD4^+^ and CD8^+^ T lymphocytes infiltrating pancreatic islets. There were no significant differences either in the percentage of CD4^+^ T cells or in CD8^+^ T cells between treated and control mice (Fig 5A). However, when CD26 expression on CD4^+^ and CD8^+^ T lymphocytes was evaluated, the MFI of CD26 expression was significantly increased in the CD8^+^ subset, but not CD4 in MK626-treated mice compared to controls (p = 0.05) (Fig 5B). These data show that, similar to what occurs with CD8^+^ T_EM_ cells from spleen and PLNs, DPPIV inhibitor MK626 increases the expression of CD26 in CD8^+^ T cells from pancreatic islet infiltrate without affecting their percentage.

### Expression of genes encoding for β-cell autoantigens after MK626 treatment

**Fig 6:**
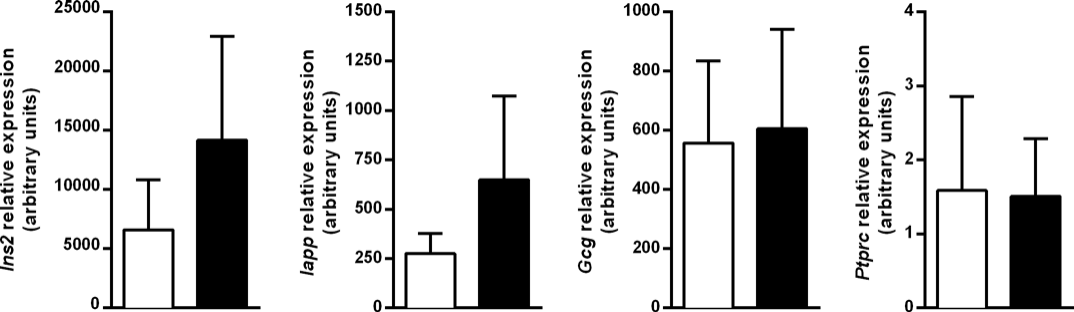
β-cell specific gene expression in the islets of MK626 NOD treated mice. Quantitative RT-PCR results for *Ins2*, *Iapp*, *Gcg and CD45* genes in control (white bars) and treated group (black bars) determined using Taqman assays. Gene expression signals were normalized to *Gadph*. All data are expressed as mean ± SD from 5 mice. Significance was tested using Mann-Whitney test.

The expression of genes encoding for *Ins2* (Insulin II) and *Iapp* (islet amyloid polypeptide) was evaluated as markers of β-cell regeneration. Specific gene expression of α-cell mass and infiltrating T lymphocytes was also determined. To that end, qRT-PCR was performed in the whole pancreases of treated mice obtained at 4 weeks after treatment. As shown in Fig 6, MK626-treatment in mice induces a tendency to increase expression of β-cell autoantigens -*Ins2* and *Iapp*- without changes in *glucagon* gene expression when compared to untreated mice. Expression levels of CD45 were similar in both groups reflecting a similar insulitis score.

### Effector memory CD8^+^ T cells from MK626-treated mice show enhanced proliferative response and cytokine production *in vitro*

**Fig 7:**
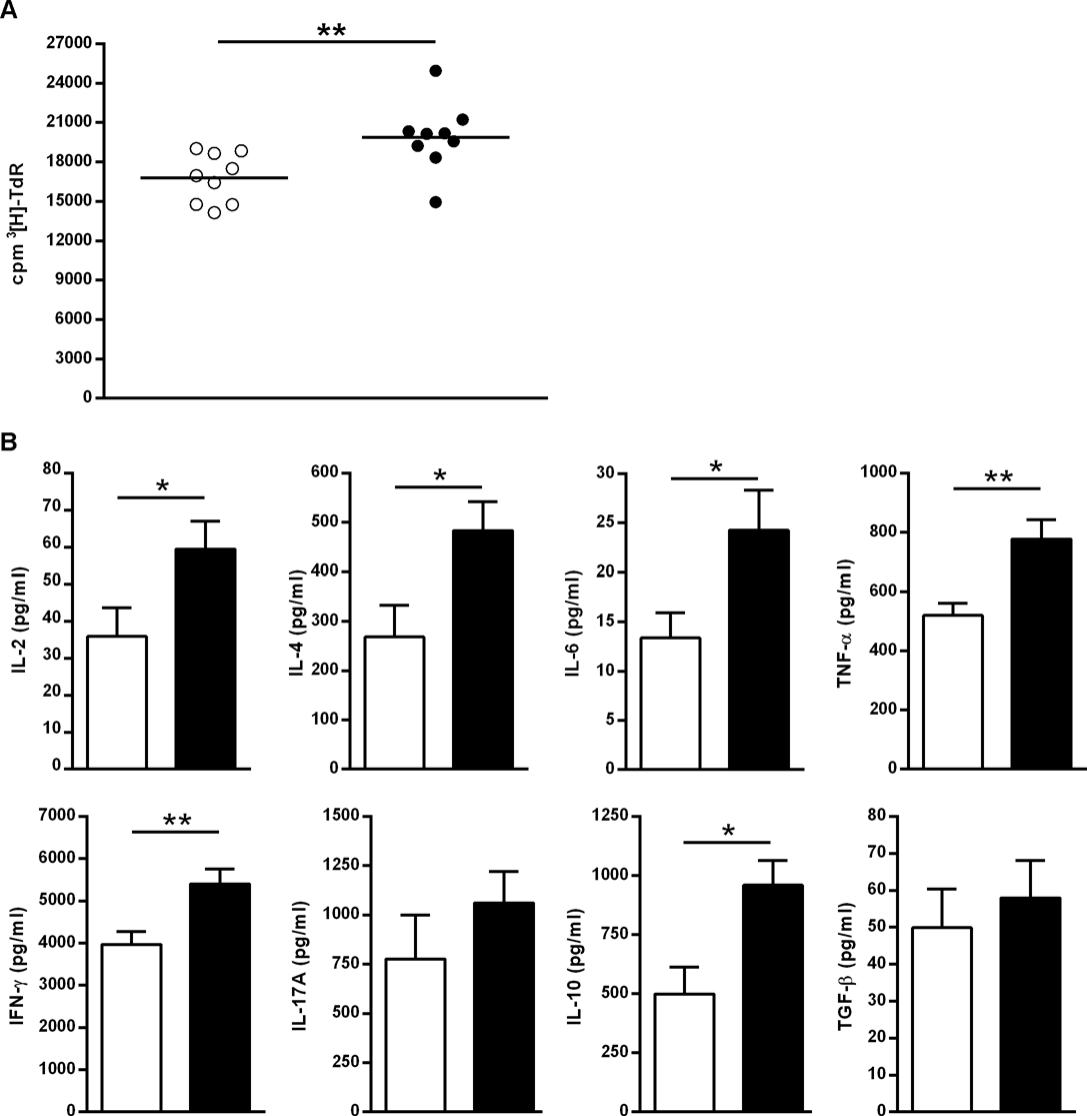
Analysis of CD8^+^ T_EM_ cell proliferation and cytokine production *in vitro* induced by MK626 treatment. CD8^+^ T_EM_ cell from control (n = 9) and treated (n = 9) mice obtained at 4 weeks of treatment were stimulated with anti–CD3 *in vitro*. A) Proliferative rate in CD8^+^ T_EM_ lymphocytes from MK626-treated mice (black circles) and in control group (white circles). T cell proliferation was expressed as counts per minute (c.p.m) using ^3^H-TdR. B) Cytokine concentration (IL-2, IL-4, IL-6, IL-10, IL-12, IL-17, IFN-γ, TNF-α and TGF-β) in supernatant from proliferation assays in treated mice (black bars) compared to control mice (white bars). Results are expressed as mean ± SEM. Mann-Whitney test was used for the evaluation of statistical significance (*p<0.05;**p<0.01).

To investigate the T cell immune responsiveness of CD8^+^ T_EM_ cells, an *in vitro* proliferation assay was performed. Purified CD8^+^ T_EM_ cells were isolated from spleen after 4 weeks of treatment and were incubated with anti-CD3. Isolated CD8^+^ T_EM_ lymphocytes from MK626-treated mice showed an increased proliferative response in comparison to the control group (p = 0.004) (Fig 7A). This CD8^+^ T_EM_ cell expansion was accompanied by a significant increase in secretion of IL-2 (p = 0.04), IL-4 (p = 0.02), IL-6 (p = 0.03), IL-10 (p = 0.01), IFN-γ (p = 0.008) and TNF-α (p = 0.005) (Fig 7B). Remarkably, IL-4, IL-10, IFN-γ and TNF-α showed the highest concentrations after stimulation. Moreover, MK626-treated mice displayed a biological reduction of IL-17 and an increase of TGF-β secretion, although statistically non-significant, when compared to control mice.

Taken together, these findings suggest that CD8^+^ T_EM_ cells from MK626-treated mice, among which are included those with a high expression of CD26, show an increased capacity of proliferative response and cytokine secretion.

### Combination of effector memory CD8^+^ T cells and MK626 treatment induces an immunosuppressive response *in vitro*

**Fig 8:**
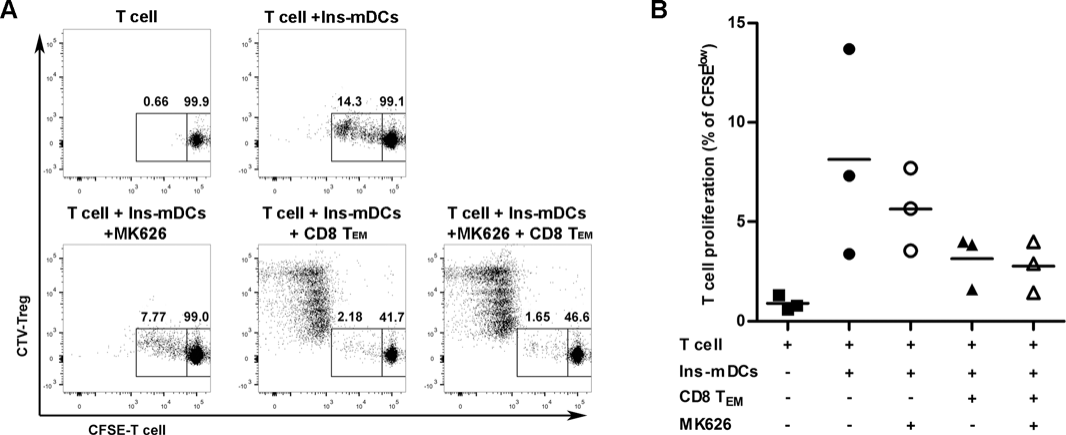
Suppressive effect of MK626 and CD8^+^ T_EM_ lymphocytes *in vitro*. A) Representative dot plots showing CFSE-T lymphocytes, stimulated with insulin-loaded mDCs, in the presence or absence of CTV-CD8^+^ T_EM_ lymphocytes (ratio 1:1) and MK626 (10μM). B) Percentage of proliferating CFSE^low^ T lymphocytes versus all T lymphocytes, with insulin-loaded mDCs (circles and triangles), in the presence of CTV-CD8^+^ T_EM_ lymphocytes (triangles) and MK626 (open symbols) for 3 days. Plots show the mean (line) of three independent experiments.

The suppressive effects of CD8^+^ T_EM_ cells on T cell proliferation was determined *in vitro*, in the presence or absence of the DPPIV inhibitor MK626. Initially, we proved that 10 μM of MK626 did not alter the viability of splenocytes (data not shown). Then, CD8^+^ T_EM_ cells were isolated to investigate the immunosuppressive ability of CD8^+^ T_EM_ cells, firstly in the absence of MK626. Despite differences did not reach statistical significance, we observed a relevant biological effect. The percentage of proliferating T cells, stimulated with insulin-loaded mDCs, was reduced by the presence of CD8^+^ T_EM_ cells at different ratios (Fig 8A and 8B). Next, when MK626 was added to co-cultures, we also observed a slight reduction in T cell proliferation at any ratio. Interestingly, the percentage of T cell proliferation also tended to reduce only in the presence of the DPPIV inhibitor MK626. Altogether, these data suggest that CD8^+^ T_EM_ cells and the DPPIV inhibitor MK626 tend to suppress antigen-stimulated T cell proliferation by themselves, and support the immunosuppressive action of CD8^+^ T_EM_ cells in the diabetogenic autoimmune response of the NOD mouse model.

## Discussion

Immunomodulation and strategies aimed at redirecting deregulated immune pathways are attractive lines of research in the field of T1D prevention. In the last decade several immune therapies have focused on limiting both islet-reactive T cell immunity and β-cell death [27–30]. The present study demonstrates that targeting CD26 with the DPPIV inhibitor MK626 reduces the incidence of T1D and insulitis degree in the pre-diabetic NOD mouse model. The analysis of peripheral and thymic T cell subsets shows that CD8^+^ T_EM_ cells are the only T cell subset affected by this treatment. More specifically, MK626 increases the expression of CD26 in CD8^+^ T_EM_ lymphocytes as well as their proliferative capacity. Furthermore, our results suggest an immunosuppressive action of CD8^+^ T_EM_ cells that could be relevant in ameliorating autoimmune diabetes through DPPIV inhibitor treatment.

In our study, DPPIV inhibition by MK626 reduces the incidence of diabetes by 31%. Prevention of T1D by targeting CD26 with the DPPIV inhibitor linagliptin has recently been demonstrated in the NOD model, with a reduction in T1D incidence of almost 50% [20]. Additionally, another DPPIV inhibitor has even been able to return hyperglycemic NOD mice to a normoglycemic state [21]. More recently, the combined treatment of low-dose monoclonal anti-CD3 antibody and the same DPPIV inhibitor used in the present study, induces the remission of diabetes in new-onset diabetic NOD mice [31].

We found that insulitis score was significantly lower in MK626-treated mice than in controls after 6 weeks of treatment. As expected, insulitis-free islets were more frequent in MK626-treated than in control mice, showing the anti-diabetogenic effect of the DPPIV inhibitor in NOD mice. The reduction of insulitis concurs with other studies conducted in NOD mice treated with DPPIV inhibitors [18,20,32]. No differences were observed in the insulitis score at 4 weeks of treatment because it still corresponds to a relatively earlier phase of the pre-diabetic state (10–12 weeks of age), whereas differences in insulitis observed at week 6 of treatment (12–14 weeks of age) may be precluding the final protective effect of DPPIV inhibitor in reducing the incidence of T1D. Potential modification of T lymphocyte migration has been proposed as an explanation for such a protective effect, and in this respect, sitagliptin has been shown to reduce T cell infiltration of grafted islets in the NOD mouse and is associated with a reduction in the *in vitro* migration of splenic and lymph node CD4^+^ T cells [19].

On the other hand, DPPIV inhibitor treatment demonstrated β-cell protective properties in different animal models of T1D [20, 33–37]. Long term administration of a DPPIV inhibitor increased the mass, replication and neogenesis of β-cells in streptozotocin-treated mice [35]. It remains unclear whether these protective effects of DPPIV inhibition on β-cell mass are mediated through incretins acting directly on β-cells increasing of endogenous GLP1 levels or through incretin-independent effects. Recently, linagliptin administrated to non-diabetic NOD mice, similarly to us, reduced insulitis, but no changes were observed in the Ki67 proliferation index of β-cells, suggesting that the increased β-cells mass observed is not an incretin-mediated effect, but rather a specific anti-inflammatory effect of DPPIV inhibition [20]. We have conducted an approach based on transcriptomics, to determine the effect of DPPIV inhibitor MK626 on β-cell regeneration. DPPIV inhibitor treatment tends to increase gene expression of β-cell autoantigens (*Ins2* and *Iapp)*, suggesting that DPPIV inhibitors may possess cytoprotective and regenerative properties. Due to the fact that no clearly significant differences in GLP-1 concentrations were obtained between MK626-treated and control mice, it may indicate that protective β-cell mass exerted by DPPIV inhibitor is based on immunomodulatory mechanisms rather than incretin effects. Similar to findings described by other authors [20], it appears that these immunoprotective effects are β-cell specific.

CD26 appears to play an important role in T cell development in the thymus. In fact, both CD26 expression and DPPIV enzymatic activity are increased during thymocyte maturation [38]. Thus, the administration of MK626 may indeed have effects on T cell development. In the present study, treatment with MK626 did not change the percentage of thymocyte subsets including SP CD4^+^, SP CD8^+^ DP and DN, nor CD26 expression between treated and control mice. However, this does not rule out that an effect of MK626 on thymocytes may eventually take place.

Regarding peripheral T cell subsets, the main finding of the present study is the increased expression of the CD26 molecule in CD8^+^ T_EM_ cell subset in treated mice. This effect was observed in the peripheral lymphocytes beginning at week 4 of treatment. In the clinical setting, the expression of CD26 has been analyzed in several peripheral blood T cell subsets in patients with T1D in which a decreased expression of CD26 in CD8^+^ [12,39] as well as in CD4^+^ T cells [39,40] have been described. These results suggest that decreased CD26 expression on several T cells subsets might represent a novel part of T lymphocyte regulatory dysfunction observed in T1D [39]. Few studies examined the effect of a DPPIV inhibitor treatment on CD26 expression in human T lymphocyte subsets. In T2D, sitagliptin administration has been associated with decreased expression of CD26 in mononuclear cells [41] and conversely, in T2D patients not on DPPIV inhibitor treatment, CD26 expression was higher in CD4^+^ and CD8^+^ T cells, although its expression was influenced by glycaemia [42]. In the present study, NOD mice were treated in a pre-diabetic stage showing normal blood glucose levels, thereby ruling out such a metabolic influence on CD26 expression.

In relation to CD8^+^ T_EM_ cells, Morimoto *et al* have recently described that human CD8^+^ T cells that express CD26 at high levels belong to the subset of early effector memory T cells [43] and studies conducted in virus infected patients suggest that these cells may be a marker of effective long-term memory T cell formation [44]. *In vitro* studies show that in humans, CD8^+^ T_EM_ cell-CD26-mediated co-stimulation has cytotoxic effects preferentially via granzyme B, TNF-α, IFN-γ and Fas ligand (FasL) and this cytotoxic function is higher than what is obtained with CD28-mediated co-stimulation.

In the present study, and in order to better characterize CD8^+^ T_EM_ subset, a cell proliferation assay was performed using an anti-CD3 antibody. We hypothesize that the increased expression of CD26 observed in this subset would account for a high proliferative rate of CD8^+^ T_EM_ cells. In this regard, it has been reported that mesotelioma cells expressing high CD26 levels display a higher proliferation activity than CD26 negative cells [45]. Results obtained in the present study show that proliferation rate was higher in CD8^+^T_EM_ cells from treated mice. Moreover, cytokine analysis from supernatant of stimulated cells with CD3-monoclonal antibody showed a higher secretion of the anti-inflammatory cytokines IL-10 and IL-4 as well as the pro-inflammatory cytokines IFN-γ and TNF-α in samples obtained from treated mice. Effector memory T cells have been described to produce higher amounts of the cytokines IL-4 and INF-γ than central memory T cells [46], in accordance with our findings. The increase in IFN-γ and TNF-α cytokines in the supernatant from CD8^+^ T_EM_ stimulated cells in the present study may be explained by the presence of CD8^+^ T_EM_ cells that express high levels of CD26 which are included in the CD8^+^ T_EM_ cell pooling treated mice, given that these cells produce high amounts of these two cytokines [43]. Moreover, levels of the anti-inflammatory cytokine IL-10 were also increased in the supernatant of stimulated CD8^+^ T_EM_ cells in treated mice, assuming that another population of non-cytotoxic T cell may be actively secreting such cytokine after MK626 plus anti-CD3. In this sense, in recent years several types of CD8^+^ T cell populations have been described as exerting a regulatory suppressive action by virtue of mainly three mechanisms: cell-to-cell contact mediated suppression, anti-inflammatory cytokine secretion and cytotoxicity to the target cells [47], some of them involving the secretion of the IL-10 [48] and some of them described as memory T cells [49,50]. In the present study, peripheral CD8 T cells were stained with the phenotypical markers CD122 and PD-1 in order to evaluate whether there were any differences in CD8^+^ Tregs between treated and control mice. No differences were observed, although results should be taken with caution given the low percentage observed of this T cell subset. Thus, this doesn’t rule out the possibility that there may exist a subset within the pool of CD8^+^ T_EM_ cells in our experiments that has immuno-regulatory properties. In fact, there is a recognized barrier while investigating CD8^+^ T suppressor cells due to the lack of a recognized specific cell marker [46,49]. Nonetheless, and despite CD8^+^ Tregs heterogenicity, there are several lines of evidence supporting the hypothesis that many Tregs subsets share a memory-like phenotype suggestive of prior antigenic encounters [51,52]. On the other hand, concerning classical Tregs such as CD4^+^CD25^+^Foxp3^+^ regulatory T cells, no increase was observed in the periphery in the present study, in agreement with Cabrera et al [32].

To further elucidate the immunoregulatory mechanism involved in the tolerance induction observed after MK626 treatment, the regulatory potential of CD8^+^ T_EM_ has been assessed in the autoimmunity context of T1D. On one hand, the *in vitro* assay shows a suppressive function of CD8^+^ T_EM_ on immune response and points to their possible anti-diabetogenic role in the NOD mouse model. On the other hand, the DPPIV inhibitor MK626 displays immunosuppressive effect by itself, according to other studies [18,53]. Due to the intrinsic suppressive potential of CD8^+^ T_EM_, we believe that MK626 might not be able to strengthen the regulatory effect of these cells *in vitro*. And, because MK626 treatment increases CD8^+^ T_EM_ proliferative capacity in our study, it is reasonable to speculate that CD8^+^ T_EM_ cells have a role in tolerance reestablishment lost in autoimmune diabetes. One way these effector cells may act is through a cytotoxic action directed against cells that are involved in antigen presentation, thereby attenuating the immune response. Additionally, a mechanism that cannot be discarded could also be related to the presence of a T cell subset within the CD8^+^ T_EM_ cell subset with immunoregulatory features due to its capacity to secrete IL-10.

Taking into account our results, future research should focus on analyzing the possible mechanisms by which CD8^+^ T_EM_ cells suppress the autoimmune response in diabetes. Although the results of the present study suggest direct immunomodulatory effects of DPPIV inhibition on T1D, a contribution of incretin modification or other additional factors that were not evaluated cannot completely be ruled out.

In conclusion, treatment with the DPPIV inhibitor MK626 reduces insulitis and decreases T1D incidence, concurring with a cytoprotective effect on β-cell mass. Furthermore, MK626 increases CD26 expression in the CD8^+^ T_EM_ subset in lymphocytes from peripheral blood, pancreatic lymph nodes and islet pancreatic infiltrate. Indeed, an immunosuppressive role for CD8^+^ T_EM_ cell subset in NOD mice may be involved in the protection against autoimmunity to β-cells. Our results provide more insights into the understanding of the mechanisms through which targeting DPPIV/CD26 reestablishes β-cell tolerance.

## Supporting Information

S1 Fig The effects of MK626 on plasma DPPIV activity and plasma active GLP-1 levels. A) Histogram of the percentage of DPPIV inhibition in control (white bars) and treated (black bars) mice after 2, 4 and 6 weeks of treatment. B) Levels of plasma active GLP-1 levels in control (white bars) and treated (black bars) mice after 2, 4 and 6 weeks of treatment; data represent mean ± SEM from 8 mice; no significant differences were found (Mann Whitney test).
(TIF)

S2 Fig Effects of MK626 on thymocytes subsets. A) Percentage of SP CD4^+^, single positive (SP) CD8^+^, double positive (DP) and double negative (DN) in untreated (white circles) and treated (black circles) mice. B) Expression (MFI) of CD26 on SP CD4^+^, SP CD8^+^, DP and DN in control (white circles) and treated (black circles) after 4 and 6 weeks of treatment. C) Percentage (left) and MFI for CD26 expression (right) on natural Tregs (CD4^+^CD25^+^FoxP3^+^) in control (white circles) and treated (black circles) mice at each checkpoint of the study. Lines represent the mean of 8 mice. Comparisons between groups did not show significant differences (three-way ANOVA).
(TIF)

S3 Fig Effects of MK626 treatment on splenic T-lymphocyte subsets from spleen after 4 and 6 weeks of treatment in control (white circles) and treated mice (black circles). A) Percentage of CD4^+^ and CD8^+^ T cells after 4 and 6 weeks of treatment in control (white circles) and treated mice (black circles). B) MFI for CD26 expression on CD4^+^ and CD8^+^ T lymphocytes after 4 and 6 weeks of treatment in control (white circles) and treated mice (black circles). C) Percentage (left) and MFI for CD26 expression (right) on Tregs (CD4^+^CD25^+^FoxP3^+^) in control (white circles) and treated (black circles) mice at each time-point of the study. Lines represent the mean of 4–10 mice. Comparisons between groups did not show significant differences (three-way ANOVA).
(TIF)
