# Supplementary figures and images for: Type 1 Diabetes Prevention in NOD Mice by Targeting DPPIV/CD26 Is Associated with Changes in CD8+T Effector Memory Subset
Source: PLoS One. 2015 Nov 10;10(11):e0142186. doi: 10.1371/journal.pone.0142186 (PMC4640511; doi:10.1371/journal.pone.0142186)

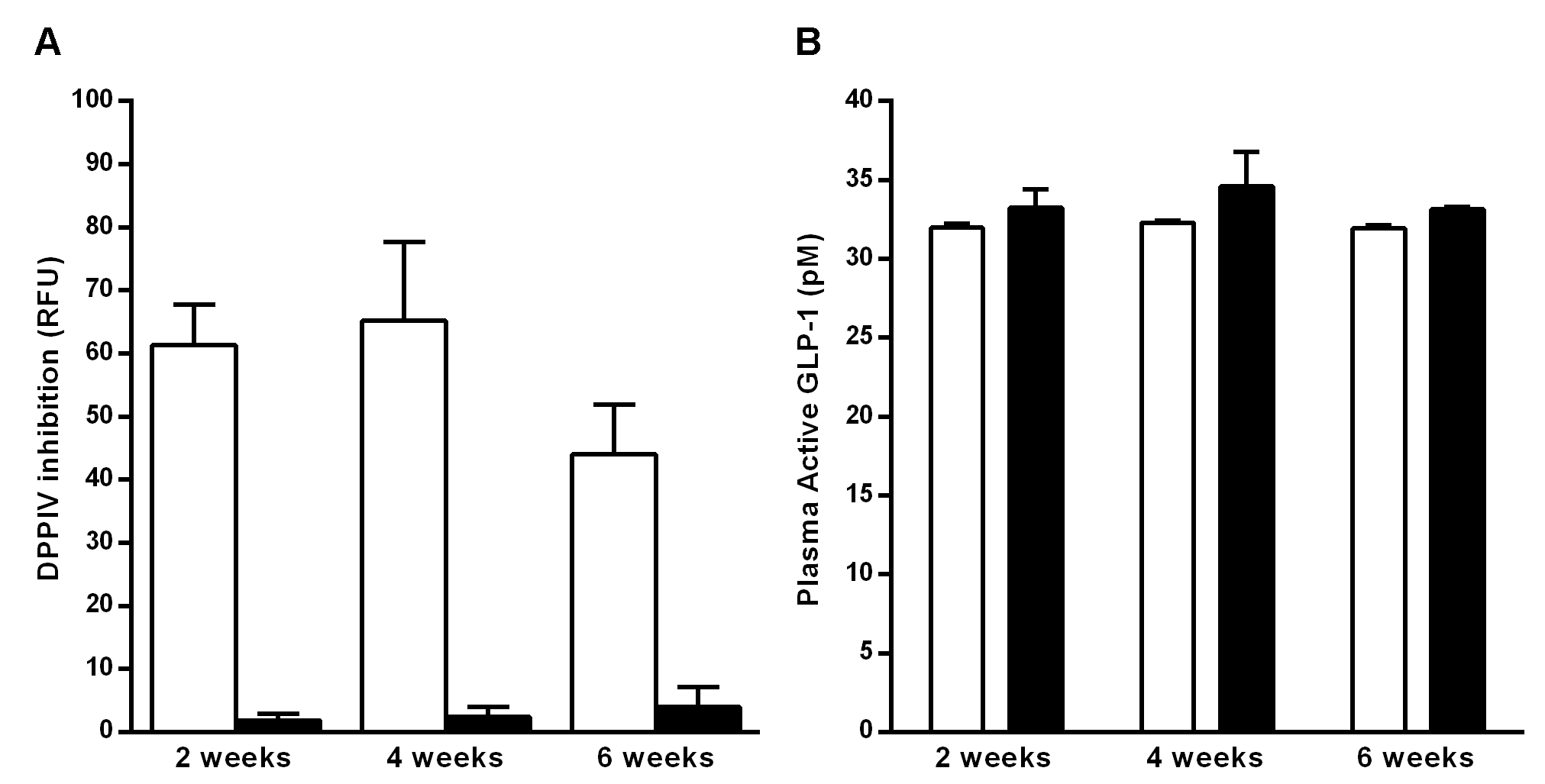

Supplement: S1 Fig — A) Histogram of the percentage of DPPIV inhibition in control (white bars) and treated (black bars) mice after 2, 4 and 6 weeks of treatment. B) Levels of plasma active GLP-1 levels in control (white bars) and treated (black bars) mice after 2, 4 and 6 weeks of treatment; data represent mean ± SEM from 8 mice; no significant differences were found (Mann Whitney test). (TIF) [file pone.0142186.s001.tif]

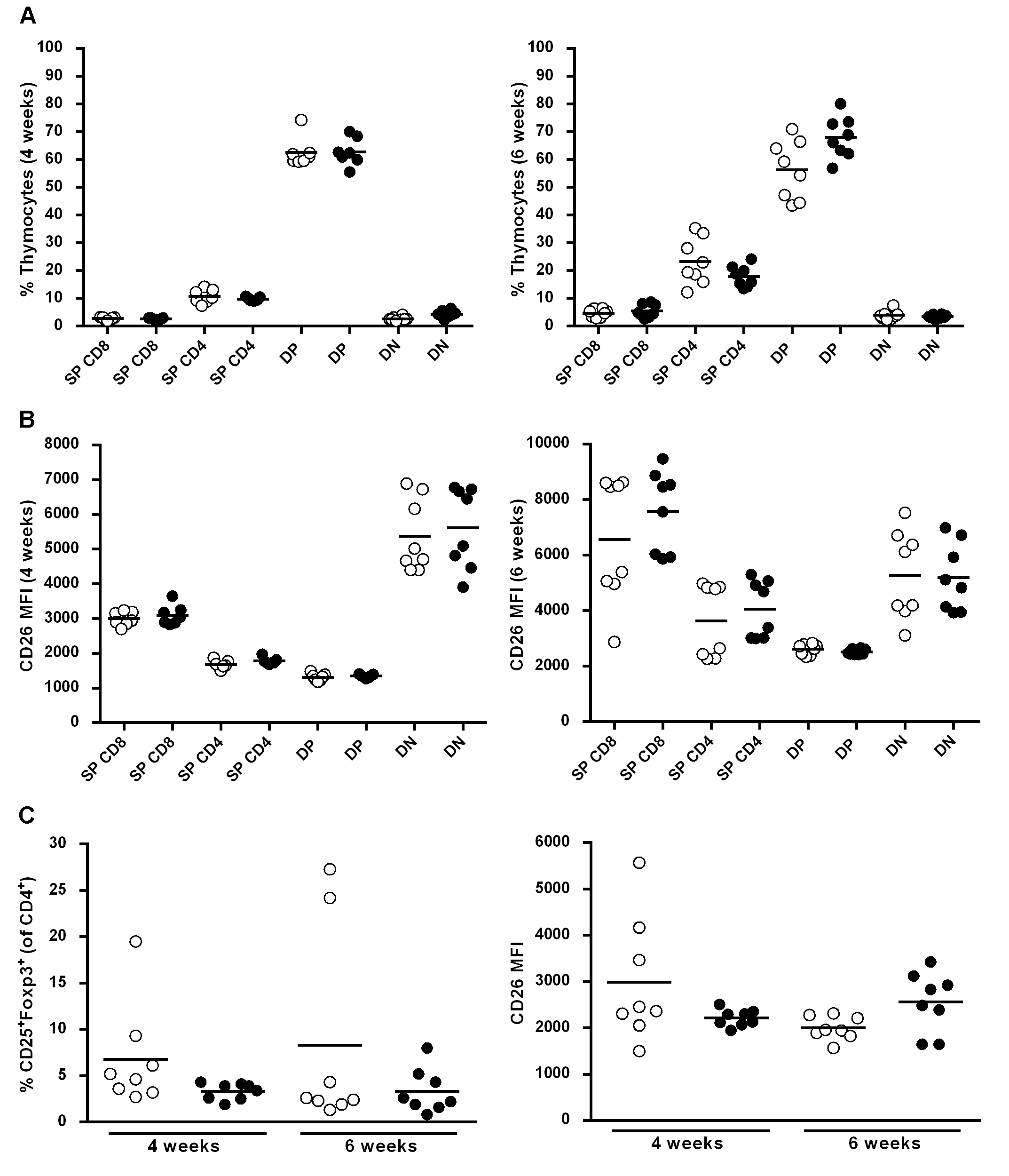

Supplement: S2 Fig — A) Percentage of SP CD4+, single positive (SP) CD8+, double positive (DP) and double negative (DN) in untreated (white circles) and treated (black circles) mice. B) Expression (MFI) of CD26 on SP CD4+, SP CD8+, DP and DN in control (white circles) and treated (black circles) after 4 and 6 weeks of treatment. C) Percentage (left) and MFI for CD26 expression (right) on natural Tregs (CD4+CD25+FoxP3+) in control (white circles) and treated (black circles) mice at each checkpoint of the study. Lines represent the mean of 8 mice. Comparisons between groups did not show significant differences (three-way ANOVA). (TIF) [file pone.0142186.s002.tif]

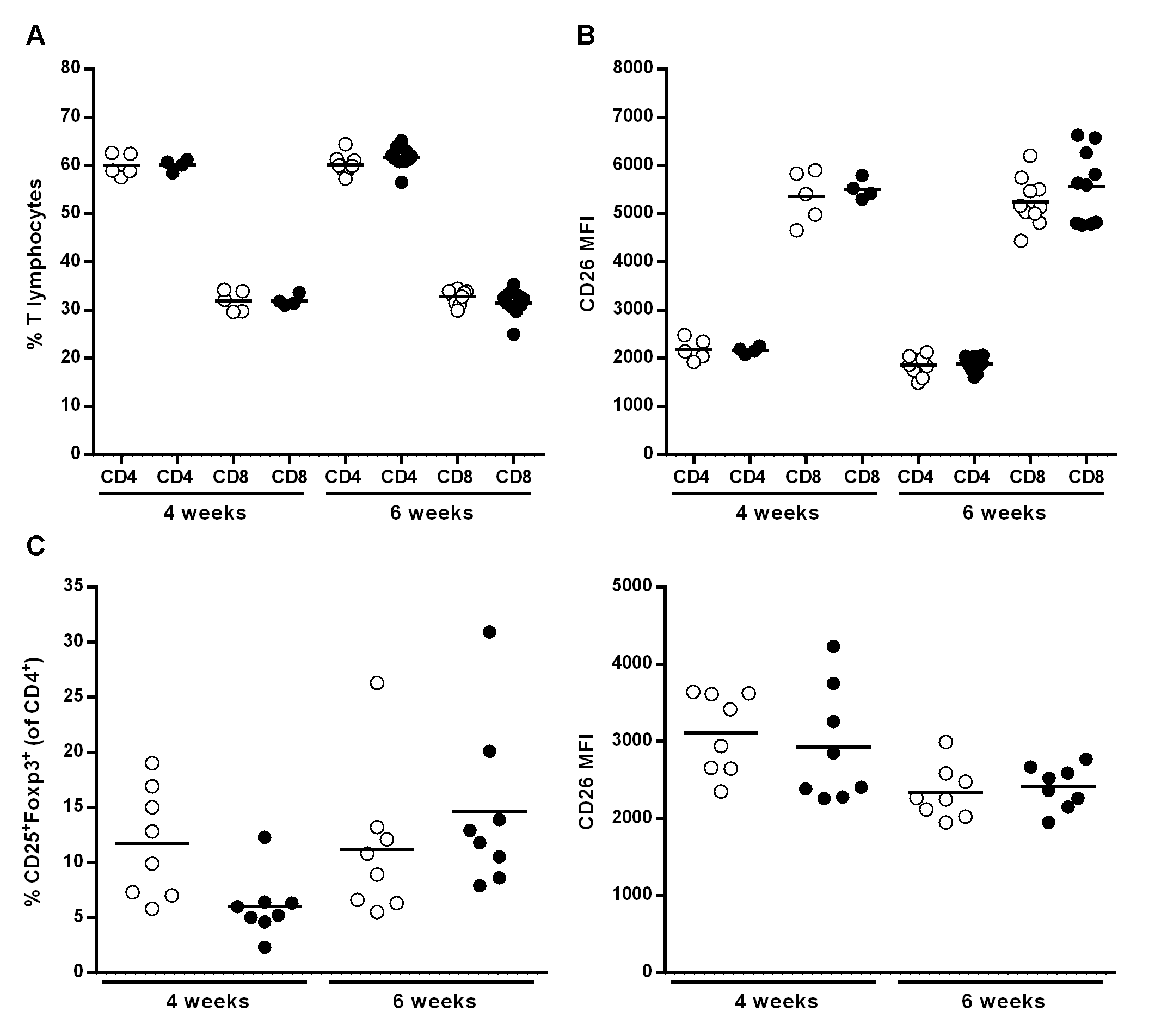

Supplement: S3 Fig — A) Percentage of CD4+ and CD8+ T cells after 4 and 6 weeks of treatment in control (white circles) and treated mice (black circles). B) MFI for CD26 expression on CD4+ and CD8+ T lymphocytes after 4 and 6 weeks of treatment in control (white circles) and treated mice (black circles). C) Percentage (left) and MFI for CD26 expression (right) on Tregs (CD4+CD25+FoxP3+) in control (white circles) and treated (black circles) mice at each time-point of the study. Lines represent the mean of 4–10 mice. Comparisons between groups did not show significant differences (three-way ANOVA). (TIF) [file pone.0142186.s003.tif]
